# Association between postoperative fall history and toe grip strength in patients after total knee arthroplasty: A prospective observational study

**DOI:** 10.1002/jfa2.12007

**Published:** 2024-04-17

**Authors:** Yuya Mawarikado, Yusuke Inagaki, Tadashi Fujii, Takanari Kubo, Akira Kido, Yasuhito Tanaka

**Affiliations:** ^1^ Graduate School of Medicine Musculoskeletal Reconstructive Surgery Nara Medical University Kashihara Nara Japan; ^2^ Department of Rehabilitation Medicine Nara Medical University Kashihara Nara Japan; ^3^ Department of Orthopeadic Surgery Kashiba Asahigaoka Hospital Kashiba Nara Japan; ^4^ Department of Rehabilitation Osaka Kawasaki Rehabilitation University Kaizuka Osaka Japan; ^5^ Department of Orthopaedic Surgery Nara Medical University Kashihara Nara Japan

**Keywords:** cohort study, falls, toe grip strength, total knee arthroplasty

## Abstract

**Background:**

Factors associated with falls after total knee arthroplasty (TKA) have been rarely reported. The aim of this study was to identify factors that influence the incidence of falls after TKA, focusing on toe grip strength (TGS) in particular, which has been associated with falls in older adults.

**Methods:**

217 patients who underwent TKA were included and followed up for 1 year. Main study outcome measures were the presence or absence of falls within 1 year after TKA. Multiple logistic regression analysis was used with postoperative falls as the dependent variable and preoperative falls and postoperative TGS on the affected sides as independent variables.

**Results:**

170 (43 and 127 in the fall and non‐fall groups) patients were included in the analysis. The presence of a preoperative falls history before TKA and a weak postoperative affected TGS indicated an increased susceptibility of the patient to fall postoperatively.

**Conclusions:**

Results of the current study revealed the association between postoperative TGS and postoperative falls. We highlight the importance of preoperative fall monitoring and postoperative TGS evaluation to prevent falls after TKA.

## 1 BACKGROUND

Total knee arthroplasty (TKA) is a surgical treatment for ameliorating pain and loss of physical function caused by knee osteoarthritis. Although TKA improves quality of life (QOL), several older adult patients may experience postoperative falls due to the high number of patients who undergo this surgery [1]. Reportedly, 12%–38% of TKA patients experience falls within 1 year after TKA [2, 3, 4]. Some reasons for this include advanced age at the time of TKA and a history of falls before TKA in approximately half of patients, which has been reported to be associated with postoperative falls [2]. Another reason is that TKA is associated with altered sensory and motor functions of the knee, which may impair balance control when standing and walking [5]. Falls after TKA are likely to result in complex fractures involving the destruction of joint implants [6]; this highlights the need for better measures to prevent falls after TKA. In the field of physical therapy, fall prevention has been considered important for maintaining QOL and preventing bed‐ridden status.

Previous studies have identified sex, age, preoperative fall history, and gait impairment as factors influencing postoperative falls after TKA [2, 7]. Similarly, another study showed that patients with a preoperative fall history were more likely to fall postoperatively [8]. Previous studies involving the general older population without knee osteoarthritis have reported that clinical factors, such as fall‐related self‐efficacy [9] and knee extension strength [10], are associated with fall risk. However, these factors may perhaps have even better predictive ability in perioperative patients scheduled to undergo TKA who generally have impaired mobility and muscle strength. Unfortunately, limited previous studies are available on falls after TKA.

Toe grip strength (TGS) is an independent risk factor for falls in older adults [11, 12]. Toe gripping, a compound movement caused by the action of the flexor hallucis brevis, flexor hallucis longus, lumbricals, flexor digitorum brevis, and flexor digitorum longus, has recently attracted attention as one of the indicators of physical function in older adults. Evidence has shown that TGS worsens with age [13, 14], thereby reducing walking speed and static balance ability [15, 16, 17]. Multiple regression analysis by Uritani et al. identified TGS as an independent factor associated with knee osteoarthritis [18], as well as a relevant factor for falls among older adults. We also reported that TGS is associated with a history of falls in patients with knee osteoarthritis prior to TKA [19].

To the best of our knowledge, no previous studies have investigated the relationship between falls and TGS in postoperative TKA patients. We hypothesized that in addition to the factors reported in previous studies, preoperative or postoperative TGS would influence falls within 1 year after TKA. In other words, we speculated that patients with greater preoperative and postoperative TGS measurements would be less likely to experience falls after TKA. If TGS is confirmed as a factor influencing falls after TKA, perioperative assessment and interventions for TKA patients would certainly be expanded. This could contribute to the possible prevention of secondary injuries, such as periprosthetic supracondylar femoral fractures. The current study aimed to analyze and identify factors that influence the incidence of falls after TKA, including TGS.

## 2 METHODS

### 2.1 Study design and participants

This study used a cohort design to identify factors influencing the presence or absence of falls 1 year after TKA. Participants were recruited from a single hospital in Japan. Participants who underwent unilateral TKA from February 2020 to May 2021 and were available for follow‐up after 1 year were recruited. The inclusion criteria were as follows: (1) participants who underwent unilateral TKA due to a diagnosis of knee osteoarthritis and were available for follow‐up 1 year after the surgery; (2) those who had the ability to ambulate independently or with a T‐cane; (3) those scheduled for primary TKA; and (4) those aged 60 years and over. The exclusion criteria were as follows: (1) patients diagnosed with rheumatoid arthritis, idiopathic osteonecrosis, or foot and ankle disorders; (2) those with bilateral toe flexion problems, neurologic, or other musculoskeletal diseases that significantly impair basic movements, such as walking; and (3) those with a diagnosis of severe depression or dementia at another hospital before TKA, which is difficult to evaluate. Those who fell at least once during the 1‐year follow‐up period after TKA were defined as the fall group, whereas those who did not fall were defined as the non‐fall group [2, 4]. The fall group was also asked about the number of falls and whether or not they were injured. For our sample size calculation, the following parameters were used: effect size = 0.5, *α* = 0.05, and power = 0.8. The allotment ratio was set to fall group/non‐fall group = 1/3 given that the postoperative fall rate was <20%–40% [2, 20]. The required sample size was determined to be 43 and 127 for the fall and non‐fall group, respectively, with an actual power of 0.804. The recruitment was closed when the number of analyzed participants reached up to 170 on schedule. Recruitment for both groups was based on the date of approval from the ethics review committee, after which participants who satisfied the eligibility criteria and did not satisfy the exclusion criteria were collected, excluding those with missing data. Recruitment was terminated when the expected number of participants in both groups was recruited.

All patients were provided with a full explanation of the study both orally and in writing in accordance with the Declaration of Helsinki, and their consent and signatures were obtained. This study was approved by the Institutional Review Board of the authors' affiliated institutions (2019‐04‐21‐007).

### 2.2 Fall definition and assessment

A fall was defined as “an event that results in a person coming to rest unintentionally on the ground or other lower level, not as a result of a major intrinsic event of overwhelming hazard” [21]. Participants self‐reported regarding the presence and frequency of falls during the year before and after TKA. To reduce the influence of recall bias, family members present at the visit were also asked about the presence and frequency of falls. Individuals who experienced 1‐year preoperative falls were interviewed the day before TKA, and those who experienced 1‐year postoperative falls were interviewed at the 1‐year postoperative follow‐up. Interviews about falls were conducted by nurses.

### 2.3 Experimental procedure

**FIGURE 1:**
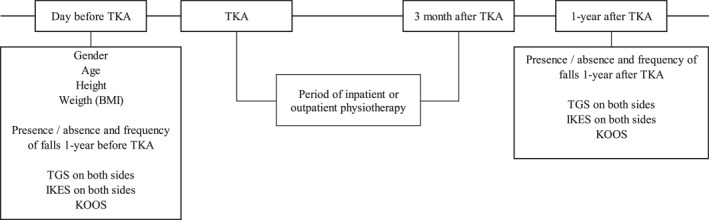
Pre‐ and post‐TKA protocols and evaluation factors. BMI, body mass index; IKES, isometric knee extension strength; KOOS, knee injury and osteoarthritis outcome score; TGS, toe grip strength; TKA, total knee arthroplasty.

Measurements were taken in a rehabilitation room and examination room. Patients were assessed a day prior to their scheduled TKA and after 1 year (Figure 1). All data were stored in the electronic medical records. The surgical side was designated as the affected side, whereas the non‐surgical side was designated as the unaffected side. TKA was performed by four orthopedic surgeons. Measurements of TGS and isometric knee extension strength (IKES) were measured by 14 randomly assigned physical therapists to reduce bias as much as possible. These physical therapists were blinded to the content and aim of the study.

#### 2.3.1 Primary outcome

**FIGURE 2:**
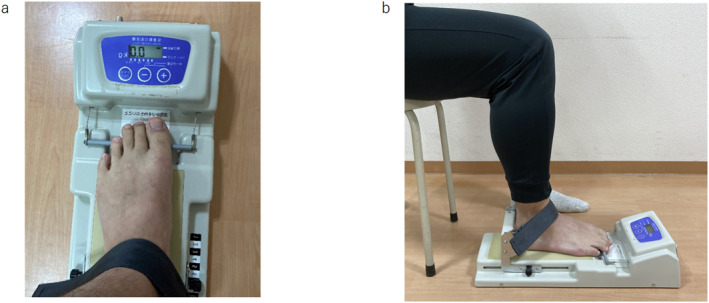
Toe grip strength (TGS) assessment. (A) Measurement of TGS: A toe grip dynamometer (T.K.K. 3361; Takei Scientific Instruments) used to measure TGS. (B) The grip bar of the instrument was adjusted to the first metatarsophalangeal joint of the participant. The participants sat on the edge of their seat keeping their trunk in a vertical position and the hip and knee joints bent to approximately 90°.

A toe grip dynamometer (T.K.K.3362; Takei Scientific Instruments) was used to measure TGS (Figure 2) in a sitting position with 90° hip and knee joint flexion and the ankle in a neutral position. The examiner adjusted the position of each participant's heel stopper so that at least the first to third toes could grasp the grip bar of the device, after which the examiner secured the foot with the provided immobilization belt to prevent it from moving. After practicing several times, the TGS of both sides was measured at the maximal isometric contractions for around 3 s. TGS was measured twice, and the mean value (kg) was calculated. Measurements were taken on the affected and unaffected sides. The inter‐ and intra‐rater reliability of this measurement protocol using the toe grip dynamometer has been found to be substantial to nearly perfect in people aged 60–79 years [22].

#### 2.3.2 Secondary outcomes

**FIGURE 3:**
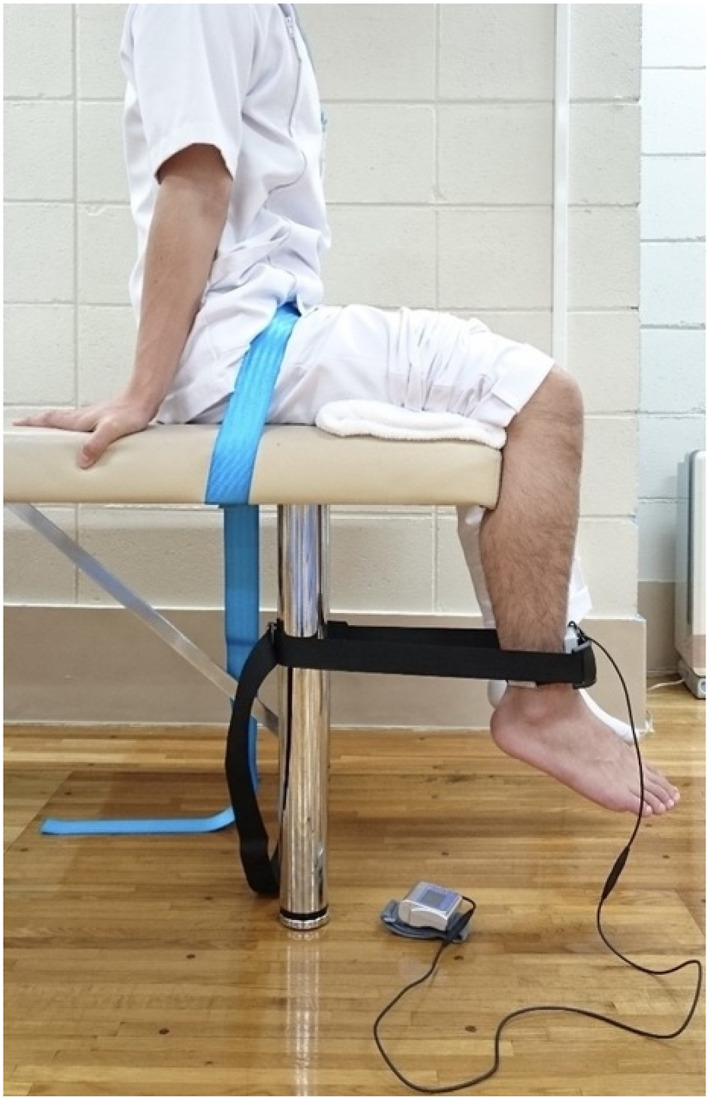
Isometric knee extension strength measurement.

Descriptive data, preoperative fall assessment (presence and frequency), IKES, and Knee Injury and Osteoarthritis Outcome Score (KOOS) were collected. Descriptive data, such as sex, age, height, weight, and body mass index, were collected by nurses who performed the measurements during the preoperative evaluation. Descriptive data were collected only before surgery. IKES was measured using a hand‐held dynamometer (μ‐tas F1, ANIMA) with participants in a seated position and the knee in 90° flexion (Figure 3) [23]. The reliability and validity of this measurement method have already been demonstrated [23, 24]. The dynamometer was placed perpendicular to the leg just above the malleoli. Participants were instructed to push against the dynamometer by attempting to straighten their knees. They were asked to gradually increase the force to maximum voluntary effort. They then maintained maximum effort for an additional 3 s. Maximum IKES was measured twice, and the mean value (kg) was calculated. Measurements were taken on the affected and unaffected sides. The KOOS has been proven to be a reliable and valid assessment tool [25]. This self‐reported questionnaire consists of 42 questions in total addressing five patient‐related domains including pain (9 questions), other disease‐specific symptoms (7 questions), activities of daily living (17 questions), sports and recreation function (5 questions), and knee‐related QOL (4 questions). Each of the five subscale scores was calculated as the sum of the factors included. The scores were converted to a 0–100 scale commonly used in orthopedics, with zero representing extreme knee problems and 100 representing no knee problems [26, 27]. Using the Likert scale, all factors had five response options ranging from 0 (no problems) to 4 (extreme problems), with scores from 0 to 100 representing the percentage of possible scores achieved.

### 2.4 Data analysis

Descriptive data were analyzed by calculating means and standard deviations or numbers and percentages. For all analyses, the significance level was set to 5%. All statistical analyses were performed using SPSS version 26.0 for Windows (SPSS Inc.). The respective preoperative and postoperative 1‐year fall rates are expressed as percentages. This also indicates how many participants who experienced falls before surgery did so after surgery. The Shapiro–Wilk test was used to determine the normality of data distribution.

To test for differences between the groups who did and did not experience postoperative falls, the unpaired *t*‐test, Mann–Whitney *U* test, and chi‐square test were used. Factors subjected to the unpaired *t*‐test were preoperative KOOS for symptoms and ADL.

The paired *t*‐test and Wilcoxon rank‐sum test were used to determine whether a significant difference between preoperative and postoperative years existed in each of the postoperative fall and non‐fall groups.

Multiple logistic regression analysis was performed to determine the effects of each factor on the presence or absence of falls within 1 year after surgery, with the presence of falls used as the dependent variable. The independent variables were preoperative fall and TGS on the affected sides after surgery. The analysis was performed using the forced entry method with age as an adjustment variable. The Hosmer–Lemeshow test was performed to determine the goodness of fit between the regression model and the actual data. The significance of the regression equation was confirmed using rate of accurate discrimination. The variance inflation factor (VIF) was calculated to account for the degree of multicollinearity among related factors.

## 3 RESULTS

### 3.1 Number of participants and fall‐related outcomes

**FIGURE 4:**
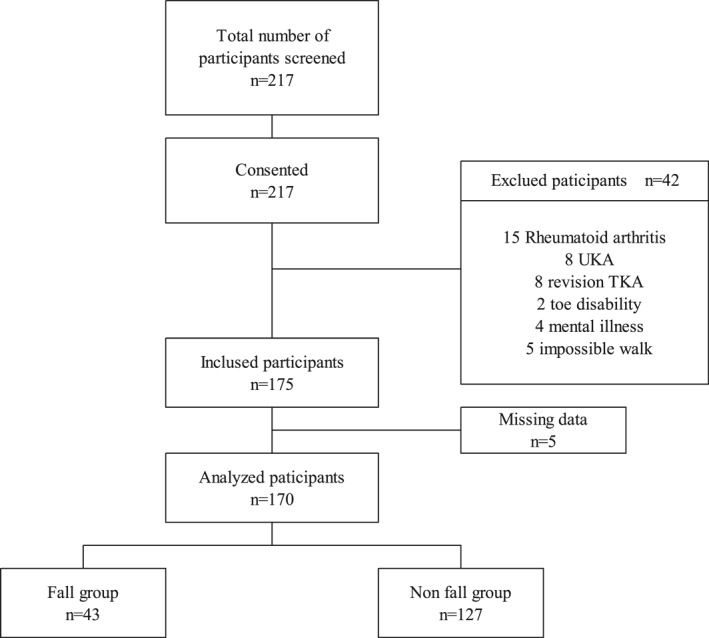
A flow diagram showing the study's inclusion criteria. TKA, total knee arthroplasty.

A flowchart for participant inclusion is shown in Figure 4. From a total of 217 participants, 170 (43 and 127 in the fall and non‐fall groups, respectively) were on schedule selected to be included in the analysis after excluding 42 participants who satisfied the exclusion criteria and another 5 with missing data. Informed consent for the study was obtained from all participants. Table 1 summarizes the participants' descriptive characteristics and the results of the unpaired *t*‐test, Mann–Whitney *U* test, and chi‐squared test. The fall rate 1 year after TKA was 25.3% (43 patients). Among the patients who suffered a fall, 28, 10, 4, and 1 reported having suffered 1, 2, 3, and 5 falls, respectively. No patients had fallen for 4 and 6 times or more. The median value of falls was 2.0 times. The fall rate 1 year before TKA was 32.4% (55 participants). Among the 55 patients who had suffered a fall before surgery, 47.3% (26 participants) had suffered a fall 1 year following TKA.

**TABLE 1:** Participants' demographic characteristics and statistical data.

	Fall group (*n* = 43)	Non fall group (*n* = 127)	*p*‐value	Phi coefficient
Gender, women (%), men	36 (83.7), 7	100 (78.7), 27	0.482	0.054
Age, years	75.51 ± 6.32	73.83 ± 7.04	0.174	
Height, cm	154.00 ± 9.42	154.27 ± 7.45	0.520	
Weight, kg	62.29 ± 11.85	63.92 ± 12.56	0.582	
BMI, kg/m^2^	26.14 ± 3.46	26.74 ± 4.19	0.798	
Preoperative fall, faller/non‐faller	26/17	29/98	0.001**	0.350
TGS, kg				
Pre‐operation				
Affected side	7.23 ± 3.49	7.41 ± 3.42	0.352	
Unaffected side	8.12 ± 3.50	7.83 ± 3.83	0.842	
1‐Year postoperatively				
Affected side	7.03 ± 2.40	8.25 ± 3.14	0.027*	
Unaffected side	7.03 ± 2.45	8.34 ± 3.26	0.025*	
IKES, kgf				
Pre‐operation				
Affected side	13.32 ± 6.42	14.04 ± 6.37	0.409	
Unaffected side	16.15 ± 6.82	16.79 ± 6.05	0.423	
1‐Year postoperatively				
Affected side	17.84 ± 8.05	19.99 ± 8.26	0.153	
Unaffected side	17.97 ± 7.42	20.42 ± 8.85	0.171	
KOOS, %				
Pre‐operation				
Symptoms	64.70 ± 18.15	59.73 ± 17.47	0.112	
Pain	50.13 ± 17.40	47.79 ± 20.01	0.407	
ADL	57.18 ± 17.66	59.98 ± 18.74	0.392	
Rec/sports	22.79 ± 22.82	22.56 ± 24.70	0.728	
QOL	30.67 ± 18.74	31.40 ± 20.10	0.891	
1‐Year postoperatively				
Symptoms	78.82 ± 15.83	81.16 ± 12.91	0.524	
Pain	82.82 ± 15.01	84.82 ± 14.50	0.346	
ADL	79.51 ± 12.99	84.26 ± 13.37	0.014*	
Rec/sports	39.30 ± 27.83	43.46 ± 30.68	0.476	
QOL	58.72 ± 21.05	61.91 ± 22.79	0.335	

*Note*: Data were expressed as mean (standard deviation), or numbers and percentages.

Abbreviations: ADL, activities of daily living; BMI, body mass index; IKES, isometric knee extension strength; KOOS, knee injury and osteoarthritis outcome score; QOL, quality of life; TGS, toe grip strength.

*p < 0.05, **p < 0.01.

### 3.2 Participant demographic characteristics and statistical data on the presence of fall

Significant differences in the number of falls (*p* = 0.001), postoperative TGS on the affected side (*p* = 0.027), postoperative TGS on the unaffected side (*p* = 0.025), and postoperative KOOS for ADL (*p* = 0.014) were observed between the fall and non‐fall groups.

### 3.3 Pre‐ and postoperative variables and statistical data for the presence of postoperative falls

Pre‐ and postoperative variables and statistical data for the presence of postoperative falls are shown in Table 2. Significant differences in TGS on the unaffected side (*p* = 0.048), the IKES on the affected side (*p* = 0.001), KOOS for symptoms (*p* = 0.001), KOOS for pain (*p* = 0.001), KOOS for ADL (*p* = 0.001), KOOS for rec/sports (*p* = 0.001), and KOOS for QOL (*p* = 0.001) were observed between the preoperative and postoperative years in the fall group.

**TABLE 2:** Pre‐ and postoperative variables and statistical data for the presence of postoperative falls.

	Preoperation	1‐Year postoperatively	*p*‐value
Fall group (*n* = 43)			
TGS, kg			
Affected side	7.23 ± 3.49	7.03 ± 2.40	0.717
Unaffected side	8.12 ± 3.50	7.03 ± 2.45	0.048*
IKES, kgf			
Affected side	13.32 ± 6.42	17.82 ± 8.05	0.001**
Unaffected side	16.14 ± 6.81	17.94 ± 7.41	0.093
KOOS, %			
Symptoms	64.70 ± 18.15	78.82 ± 15.83	0.001**
Pain	50.13 ± 17.40	82.82 ± 15.01	0.001**
ADL	57.18 ± 17.66	79.51 ± 12.99	0.001**
Rec/sports	22.79 ± 22.82	39.30 ± 27.83	0.001**
QOL	30.67 ± 18.74	58.72 ± 21.05	0.001**
Non fall group (*n* = 127)			
TGS, kg			
Affected side	7.41 ± 3.42	8.25 ± 3.14	0.012*
Anaffected side	7.83 ± 3.83	8.34 ± 3.26	0.043*
IKES, kgf			
Affected side	14.22 ± 6.41	20.01 ± 8.26	0.001**
Unaffected side	16.79 ± 6.05	20.42 ± 8.85	0.001**
KOOS, %			
Symptoms	59.73 ± 17.47	81.16 ± 12.91	0.001**
Pain	47.79 ± 20.01	84.82 ± 14.50	0.001**
ADL	59.98 ± 18.73	84.26 ± 13.37	0.001**
Rec/sports	22.56 ± 24.70	43.46 ± 30.68	0.001**
QOL	31.40 ± 20.10	61.91 ± 22.79	0.001**

Abbreviations: ADL, activities of daily living; IKES, isometric knee extension strength; KOOS, knee injury and osteoarthritis outcome score; QOL, quality of life; TGS, toe grip strength.

*p < 0.05, **p < 0.01.

Significant differences in TGS on the affected side (*p* = 0.012), IKES on the affected (*p* = 0.001) and unaffected side (*p* = 0.001), KOOS for symptoms (*p* = 0.001), KOOS for pain (*p* = 0.001), KOOS for ADL (*p* = 0.001), KOOS for rec/sports (*p* = 0.001), and KOOS for QOL (*p* = 0.001) were observed between the preoperative and postoperative years in the non‐fall group.

### 3.4 Factors affecting falls during the 1‐year after surgery in the fall group

The results for multiple logistic regression analysis are shown in Table 3. Accordingly, the model chi‐squared test (significant at *p* < 0.01) showed that preoperative falls (*B* = 1.659, *p* = 0.001, odds ratio (OR) = 5.253) and postoperative TGS on the affected side (*B* = −0.150, *p* = 0.040, OR = 0.860) were significantly associated with postoperative falls. The result of the Hosmer–Lemeshow test was not significant at *p* = 0.384, and the fit of the regression equation was good. The rate of accurate discrimination was 76.5%. The VIF for preoperative falls and TGS on the affected side were both 1.003, with no multicollinearity observed.

**TABLE 3:** Multiple logistic regression analysis for the presence of postoperative falls.

	*B*	Standard error	Wald	*p* value	Odds ratio (95% confidence interval)
Preoperative fall	1.659	0.387	18.364	0.001	5.253 (2.460–11.216)
Postoperative TGS on the affected side	−0.150	0.073	4.233	0.040	0.860 (0.746–0.993)
Age	0.027	0.029	0.898	0.343	1.028 (0.971–1.087)
Constant	−2.655	2.347	1.279	0.258	

*Note*: Regression equation: ln(*p*/1 − *p*) = 1.659 (preoperative fall) − 0.150 (TGS on the affected side) + 0.027 (age) − 2.655 model *x* ^2^, *p* < 0.01; Hosmer–Lemeshow test, *p* = 0.394; rate of accurate discrimination, 76.5%.

Abbreviation: TGS, toe grip strength.

## 4 DISCUSSION

The current study aimed to identify pre‐ and postoperative factors that influenced the presence or absence of falls after TKA, which could facilitate future interventions and patient education. To the best of our knowledge, this has been the first study to investigate the relationship between falls and TGS in patients after TKA. Our findings showed that 55 (32.4%), and 43 (25.3%) patients suffered falls 1 year before and after surgery, respectively. Similar to previous studies in other countries, we found that fall rates were higher before than after surgery [3, 4]. This seems to suggest that after TKA, surgeons and physical therapists often inform their patients regarding their risk for falls. Patient education in falls prevention provided by healthcare professionals may also play a role in decreasing the number of falls after TKA [28, 29]. Perhaps the decrease in falls after TKA is associated with not only pain and lower extremity muscle strength but also patients' increased attention to falls.

Multiple logistic regression analysis revealed that in addition to preoperative fall history, TGS on the affected side was a factor influencing falls 1 year after surgery. To our knowledge, this has been the first report to show that TGS had an influence on falls after TKA. Many studies reported an association between TGS and falls in older adults. With increasing age, TGS in older adults deteriorates [13, 14, 30], resulting in decreased walking speed and static balance ability [15, 16, 17]. Tsuyuguchi et al. recruited middle‐aged adults and, whose average age was 62.02, divided them into high and low risk of falls. They found TGS to be an independent risk factor for fall occurrence [31]. Although the causal relationship still remains unclear, we believe that TGS contributes to the challenges in patients with knee osteoarthritis who frequently fall. The current study showed that postoperative TGS, but not preoperative TGS, influenced postoperative falls. Considering the study by Mawarikado et al. [19], we can surmise that preoperative falls were influenced by preoperative TGS, whereas postoperative falls were influenced by postoperative TGS. As such, we believe that the impact of TGS on perioperative falls in TKA patients cannot be examined longitudinally; instead, TGS must be evaluated before and after surgery to determine the risk for pre‐ and postoperative fall events, respectively. The advantage is that TGS is outside the affected parts of the TKA, enabling intervention in the early postoperative period.

Our chi‐squared test results showed that a preoperative fall history was associated with postoperative falls. Moreover, multiple logistic regression analysis identified preoperative falls as the strongest factor associated with postoperative falls. It is unsurprising that older adult patients who had experienced falls before TKA were more likely to suffer the same after the procedure, as suggested in previous studies [32]. Swinkels et al. [2] found that postoperative TKA patients showed improvement in function and balance up to 1 year after surgery. However, those with a history of falls before surgery showed no improvement in balance after surgery and no improvement in balance or function 1 year after TKA. We believe that regardless of whether TKA provides pain relief and functional improvements in patients with a preoperative risk for falls, multiple other factors may influence and even increase the risk for falls.

The current study has several limitations worth noting. First, given that the fall survey was conducted 1 year after surgery, we could not determine specifically whether the fall occurred immediately after discharge from the hospital or at 6 months to 1 year after surgery. In the future, TGS and other assessment items should be evaluated periodically. Second, various factors have been suggested to influence a patient's risk for falls, emphasizing knee arthropathy in patients undergoing TKA should be considered holistically, particularly with regard to its functional and clinical impairments and its interaction with visual and vestibular function, rather than simply as a joint‐related disease. However, given that arthroplasty is a joint replacement procedure for arthropathy, we also believe it important to consider specific assessment factors in the perioperative period. Third, the influence of multiple risk factors, such as polypharmacy [33], visual impairment [34] and depression [35], is not taken into account. Those risk factors are associated with lower fall rates or fall risk in older adults [33, 36]. In the future, we believe that the risk of falls after TKA should be considered, including other risk factors. Fourth, there was no break between the practice repetitions and the actual trials of TGS and IKES. Potential confounding effects of fatigue in the two measured outcomes could not be prevented. The final point is the lack of assessment on dominant and non‐dominant sides. TGS of the dominant foot has been identified as a risk factor for falls in older people [37]. By asking for lower limb dominance, we can easily predict falls.

## 5 CONCLUSIONS

This current study investigated the relationship between TGS and presence or absence of falls within 1 year after TKA. Our results showed that the presence of falls after TKA was associated with the presence of falls before surgery and the intensity of postoperative TGS. A novel finding of our study was that postoperative TGS may influence postoperative falls. This study takes the first step in demonstrating the significance of evaluating TGS to determine the risk for falls after TKA. Considering our findings suggesting that TGS affects falls before [19] and after TKA, the next step would be to determine how TGS training affects falls.

## AUTHOR CONTRIBUTIONS

*Conceptualization*: Yuya Mawarikado, Yusuke Inagaki, Akira Kido, and Yasuhito Tanaka. *Data curation*: Yuya Mawarikado and Yusuke Inagaki. *Formal analysis*: Yuya Mawarikado and Yusuke Inagaki. *Investigation*: Yuya Mawarikado, Yusuke Inagaki, Takanari Kubo, and Tadashi Fujii. *Methodology*: Yuya Mawarikado, Yusuke Inagaki, Takanari Kubo, and Tadashi Fujii. *Project administration*: Yuya Mawarikado. *Resources*: Yuya Mawarikado and Tadashi Fujii. *Software*: Yuya Mawarikado and Yusuke Inagaki. *Visualization*: Yuya Mawarikado and Yusuke Inagaki. *Writing—original draft*: all authors. *Writing—review and editing*: all authors.

## CONFLICT OF INTEREST STATEMENT

The authors declare no conflicts of interest.

## ETHICS STATEMENT

This study was approved by the Institutional Review Board of the authors' affiliated institutions. This study complied with the Declaration of Helsinki. Details of the study protocol and the aim of the study were explained to all participants and written informed consent was obtained from all participants before including them in the study.

## CONSENT FOR PUBLICATION

Not applicable.

## Supporting information

Supplementary Information S1

## Data Availability

The data that support the findings of this study are available from the corresponding author upon reasonable request.
